# The silent predictors: exploring galectin-3 and Irisin’s tale in severe COVID-19

**DOI:** 10.1186/s13104-024-06978-3

**Published:** 2024-10-27

**Authors:** Valentina N. Nikolic, Višeslav Popadic, Slobodan M. Jankovic, Nenad Govedarović, Stevan Vujić, Jelica Andjelković, Lazar S. Stosic, Nikola Č. Stevanović, Marija Zdravkovic, Zoran Todorovic

**Affiliations:** 1 Department of Pharmacology with Toxicology, University of Nis Faculty of Medicine, Bul. dr Zorana Djindjica 81 Nis, Nis, 18000 Serbia; 2 University Hospital Medical Center, Bezanijska kosa, Belgrade, Serbia; 3 Department of Pharmacology and Toxicology, Faculty of Medical Sciences, University of Kragujevac, Kragujevac, Serbia; 4 Department of Internal Medicine, University of Nis Faculty of Medicine, Nis, Serbia; 5 University of Nis Faculty of Medicine, Nis, Serbia; 6 Department of Internal Medicine, Faculty of Medicine, University of Belgrade, Belgrade, Serbia; 7 Department of Pharmacology, Clinical Pharmacology and Toxicology, Faculty of Medicine, University of Belgrade, Belgrade, Serbia

**Keywords:** COVID-19 prognosis, Galectin-3, Irisin, SARS-CoV-2 infection, Biomarker discovery

## Abstract

**Objective:**

This study aimed to evaluate the roles of galectin-3 and irisin as biomarkers in predicting severe outcomes in COVID-19 patients.

**Results:**

We analyzed serum levels of galectin-3 and irisin in 59 patients with severe COVID-19 and 30 healthy controls. Elevated galectin-3 levels were associated with increased risks of mortality, need for intensive care, and severe acute respiratory distress syndrome. The optimal cut-off value for galectin-3 was 13.47 ng/ml, with a sensitivity of 72.7% and specificity of 76.6%. Irisin levels did not differ significantly between survivors and non-survivors at admission or on the 3rd day post-admission, but approached significance on the 7th day. These findings suggest that galectin-3 could be a valuable prognostic biomarker for severe COVID-19 outcomes, while irisin’s role remains to be clarified in further studies.

## Introduction

Since its emergence in late 2019, the COVID-19 pandemic caused by SARS-CoV-2 has become a major global health crisis. It has affected over 772 million people and caused nearly 7 million deaths worldwide [1]. The severity of COVID-19 varies widely, often exacerbated by the host’s immune response, including cytokine storms and acute respiratory distress syndrome (ARDS) [2, 3]. This variation underscores the critical need for reliable biomarkers to predict disease progression, especially in patients with underlying metabolic issues like insulin resistance, obesity, and diabetes.

Insulin resistance, common in obesity and type 2 diabetes mellitus (T2DM), exacerbates COVID-19 outcomes [4, 5]. When combined with SARS-CoV-2 infection, this resistance fosters a proinflammatory state that further disrupts glucose metabolism. Irisin, a myokine induced by physical exercise, has emerged as a promising biomarker for severe COVID-19 outcomes [6, 7]. Investigating irisin levels in COVID-19 patients could provide crucial insights for identifying those at increased risk and developing targeted interventions.

Concurrently, galectin-3 (Gal-3), a lectin involved in fibrosis, inflammation, and immune regulation, is being closely studied [8]. Recent research highlights the effectiveness of Gal-3 inhibitors, such as inhaled GB0139 and orally administered ProLectin-M (PL-M), in modulating immune responses and reducing severe COVID-19 inflammation [9]. The success of these inhibitors in decreasing viral load and alleviating symptoms suggests Gal-3 as a potential target for treating severe COVID-19 cases.

Our study assesses serum irisin and galectin-3 levels as prognostic biomarkers for mortality risk in severe COVID-19 patients. We aim to demonstrate their effectiveness in identifying high-risk cases and improving patient management strategies. By understanding the roles of irisin and galectin-3, we enhance treatment approaches and outcome predictions for severe SARS-CoV-2 infections.

## Materials and methods

### Study design and participant selection

This prospective study, conducted under the approval of the Ethics Committee of the University Clinical Hospital Center Bezanijska kosa, Belgrade, Serbia (decision number 1047/2) and adhering to the Helsinki Declaration, enrolled adults diagnosed with severe COVID-19 confirmed via RT-PCR. Exclusion criteria included pregnancy, immunosuppressive therapy, or recent severe acute conditions. Clinical assessments at admission included chest radiography and CT scans, essential for assessing COVID-19 pneumonia severity, with subsequent scans for patients showing clinical deterioration. Treatment protocols followed national guidelines and WHO recommendations.

### Data collection

Comprehensive demographic, clinical, and laboratory data were collected through medical records and the hospital’s health information system (Heliant, v7.3, r48602). Collected data encompassed age, gender, Body Mass Index - BMI, medical history (including hypertension, diabetes mellitus, chronic obstructive pulmonary disease - COPD), and laboratory values (interleukin-6 - IL-6, C-reactive protein - CRP, procalcitonin, ferritin, D-dimer, serum albumin, lymphocyte count, platelet count, prothrombin time, activated partial thromboplastin time, and fibrinogen), alongside CT severity scores.

### Biomarker measurement

Serum irisin and galectin-3 levels were measured using ELISA kits from FineTest Corp. (Cat.No: EH4702 for irisin and EH0145 for galectin-3), per the manufacturer’s protocols. Blood samples for serum irisin were collected at admission, and on the 3rd and 7th days post-admission to monitor its dynamic levels, reflecting disease progression. Galectin-3 sampling coincided with routine laboratory analyses at admission.

### Statistical analysis

Numerical data were presented as means ± standard deviations or medians with interquartile ranges, and categorical data in absolute numbers and percentages. Statistical comparisons between survivors and non-survivors were performed using Student’s t-test, Mann–Whitney U-test, and Chi-square test. Predictors of mortality were identified through univariate and multivariate logistic regression, with results expressed as odds ratios and 95% confidence intervals. The model’s performance was evaluated using sensitivity, specificity, and the area under the ROC curve. Statistical analyses utilized IBM SPSS Statistics software version 25.0.

## Results

### Patient demographics and clinical characteristics

The study involved 59 patients with severe COVID-19 and 30 healthy controls. The COVID-19 group was further divided based on in-hospital outcomes into survivors and nonsurvivors. While there were no significant differences in age, gender, or (BMI) between these groups (*p* > 0.05 for all), significant differences in inflammatory markers such as CRP, IL6, and D-dimer were found, significantly elevated in nonsurvivors (see Table 1).

**Table 1:** Characteristics of the study groups

Variable	Cured and discharged from a hospital (*n* = 48)	Died (*n* = 11)	*p* value
Age (years)	67.5 ± 11.2, 69 [14]	70.4 ± 10.2, 74 [9]	0.288
Sex (m/f)	20/28 (41.7%/58.3%)	4/7 (36.4%/63.6%)	1.000
BMI > 30	26/22 (54.2%/45.8%)	6/5 (54.5%/45.5%)	0.982
Maximum CRP value during hospitalization	86.5 ± 69.6, 79.2 [101.2]	216.7 ± 97.4, 261.5 [157.3]	0.000
IL6	64.3 ± 115.7, 26.4 [64.2]	211.5 ± 249.1, 118.7 [360.8]	0.005
D-dimer	1871.7 ± 3504.6, 880.0 [1090]	8341.9 ± 7577.2, 4288.0 [12373]	0.000
Minimum albumin level during hospitalization	34.4 ± 4.3, 35.0 [4.0]	25.3 ± 4.5, 26.0 [6.0]	0.000
Minimum lymphocyte level during hospitalization (x 10^9^/L)	0.8 ± 0.4, 0.7 [0.6]	0.3 ± 0.2, 0.3 [0.3]	0.000
Length of hospitalization	12.8 ± 6.4, 11.0 [5.0]	18.4 ± 8.6, 16.0 [15.0]	0.040
CT score	10.9 ± 5.0, 10.0 [8.0]	16.4 ± 6.0, 15.0 [11.0]	0.028
Artificial ventilation (yes/no)	0/48 (54.2%/45.8%)	5/6 (45.5%/54.5%)	0.000
HTA (yes/no)	33/15 (68.8%/31.2%)	7/4 (63.6%/36.4%)	0.734
Diabetes (yes/no)	17/31 (35.4%/74.6%)	5/6 (45.5%/54.5%)	0.535
COPD (yes/no)	1/47 (2.1%/97.9%)	2/9 (18.2%/81.8%)	0.059
Asthma (yes/no)	2/46 (4.2%/95.8%)	1/10 (9.1%/90.9%)	0.468
Coronary disease (yes/no)	8/40 (16.7%/83.3%)	4/7 (36.4%/63.6%)	0.209
Cardiomyopathy (yes/no)	5/43 (10.4%/89.6%)	2/9 (18.2%/81.8%)	0.604
Renal failure (yes/no)	1/47 (2.1%/97.9%)	1/9 (10.0%/90.0%)	0.318
Irisin on admission	418.1 ± 132.4, 458.3 [187.3]	395.0 ± 148.4, 471.2 [273.0]	0.992
Irisin 3rd day	446.6 ± 241.0, 373.0 [425.3]	358.4 ± 164.7, 304.1 [325.3]	0.406
Irisin 7th day	289.3 ± 167.2, 254.7 [318.9]	385.3 ± 142.9, 380.8 [298.1]	0.097
Galectin on admission	11.5 ± 3.6, 11.5 [5.1]	14.3 ± 3.9, 14.8 [4.7]	0.019

* Categorical variables were compared by Chi square test, or Fisher test when cell counts were below 5; continuous variables were compared by Student’s T test when normally distributed, or by Mann Whitney U test, when the distribution was not normal

### Serum biomarker levels

**Fig. 1:**
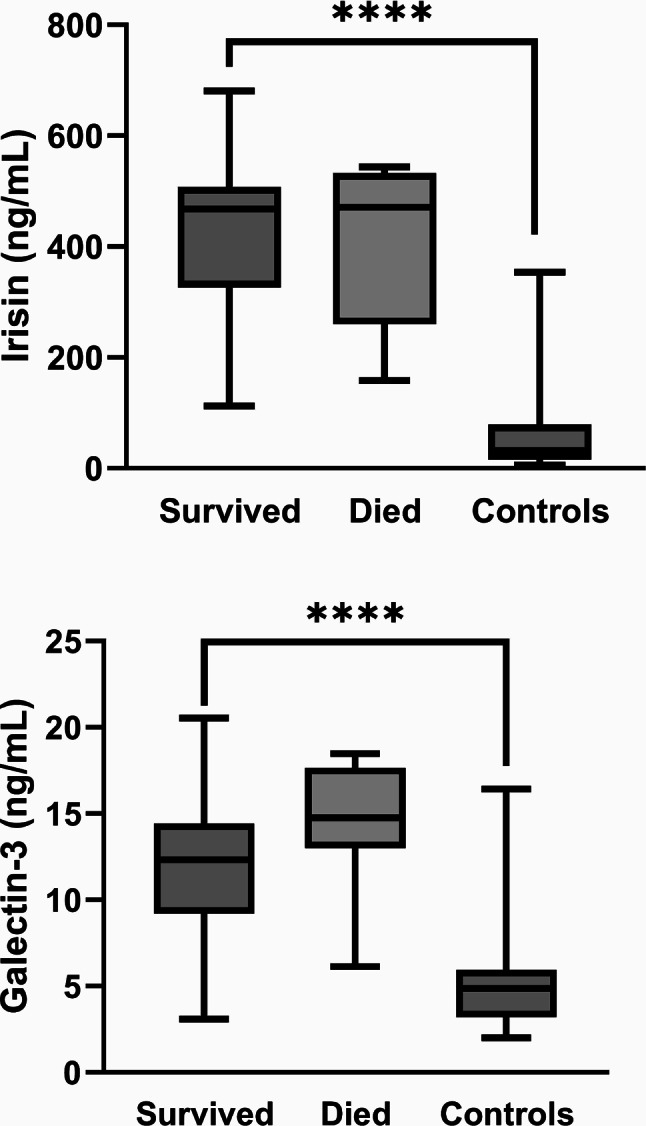
Galectin-3 level variations across different study cohorts

Analysis of serum biomarker levels revealed that galectin-3 was significantly higher in nonsurvivors (14.3 ± 3.9 ng/ml) compared to survivors (11.5 ± 3.6 ng/ml) and controls (5.2 ± 2.9 ng/ml), suggesting its importance as a prognostic biomarker (*p* = 0.019). This differential expression highlights galectin-3’s potential role in predicting severe outcomes in COVID-19 cases. For a visual representation of these differences among the groups, see Fig. 1. The analysis of irisin levels at different time points showed that while there was no statistically significant difference in irisin levels on admission and the 3rd day post-admission between survivors and non-survivors, the levels on the 7th day post-admission approached statistical significance (*p* = 0.097). This suggests that with a slightly larger sample size, a significant difference might have been observed.

### Predictive value of galectin-3

**Fig. 2:**
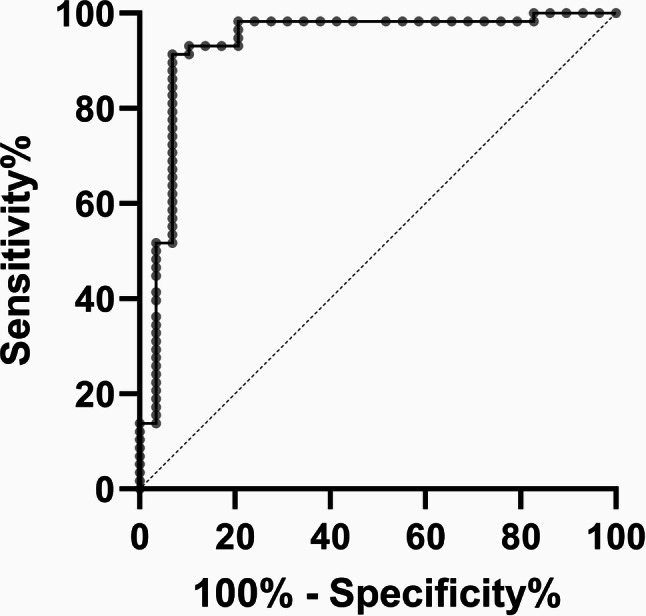
ROC curve analysis for galectin-3 as a predictor of mortality in patients with severe COVID-19

Multivariate logistic regression confirmed galectin-3 as an independent predictor of mortality in severe COVID-19 cases, with each unit increase in serum level significantly associated with an increased risk of mortality risk (*p* = 0.037). ROC curve analysis supported the robustness of galectin-3’s predictive value, demonstrating an Area Under the Curve (AUC) of 0.729, indicative of good predictive accuracy. The sensitivity and specificity of this biomarker for predicting mortality were established at 72.7% and 76.6%, respectively. From these analyses, a critical cut-off value for galectin-3 was determined to be 13.47 ng/ml. Patients with levels above this threshold are considered to be at a significantly higher risk of severe outcomes. This biomarker’s effectiveness underscores its potential utility in clinical settings for risk stratification and guiding management strategies in severe COVID-19 cases. Detailed statistical results can be found in Table 2, and the predictive performance of galectin-3 is visually represented in Fig. 2, which displays the ROC curve.

**Table 2:** Uni-and multi-variate logistic regression* analysis with death as an outcome

Predictor	Crude ODDs ratio (95%CI)	*p* value	Adjusted ODDs ratio (95%CI)	*p* value
Galectin serum concentration	1.242 (1.015–1.520)	0.035	1.267 (1.015–1.583)	0.037**
Diabetes mellitus	1.520 (0.403–5.723)	0.536	1.329 (0.288–6.141)	0.715
COPD	10.444 (0.854–127.768)	0.066	15.787 (0.973–256.077)	0.052
Cardiomyopathy	1.911 (0.319–11.450)		2.375 (0.336–16.788)	0.386

* multivariate logistic regression model was built using the best subsets regression method. Omnibus test was significant (*p* = 0.045), Hosmer Lemeshow test showed that the observed event rates matched the expected event rates in the population subgroups (*p* = 0.445), Cox & Snell R square was 0.155, and Nagelkerke Rsquare was 0.249

** significant effect

#### Comparative analysis

Galectin-3 showed a stronger correlation with mortality outcomes in severe COVID-19 patients compared to traditional clinical and laboratory markers. This underscores its potential utility in enhancing patient management strategies, especially in guiding early interventions based on risk stratification.

## Discusion

Our study confirms galectin-3 (Gal-3) as a crucial biomarker for predicting severe outcomes in COVID-19, including increased mortality and the development of severe acute respiratory distress syndrome (ARDS).

Gal-3 enhances the virus’s inflammatory effects by promoting cytokine release from human monocytes (Schroeder et al. [10]). This relationship contributes to severe disease manifestations such as ARDS and increased mortality rates ([11–14]).

Several mechanisms may explain why elevated Gal-3 levels are associated with increased mortality and severe outcomes in COVID-19 patients. Elevated Gal-3 levels contribute to the hyperinflammatory response observed in severe COVID-19 cases. Gal-3 upregulates the production of pro-inflammatory cytokines such as IL-6 and TNF-α, critical drivers of the cytokine storm, leading to severe tissue damage and organ failure [8]). Additionally, Gal-3 plays a significant role in the development of fibrosis. In the context of COVID-19, Gal-3 promotes the activation of fibroblasts and macrophages, leading to lung fibrosis, a common consequence of the prolonged inflammatory response in severe cases, contributing to the deterioration of lung function and poor clinical outcomes (15). Gal-3 can also enhance viral entry and infection. It has been suggested that Gal-3 may facilitate the binding of the SARS-CoV-2 spike protein to host cell receptors, thereby promoting viral entry. Gal-3’s interaction with other receptors, such as CD147 and CD26, further supports the viral infection, leading to more severe disease manifestations [10].

A prospective cohort study involving 156 patients demonstrated Gal-3’s high predictive accuracy (AUC of 0.85) in distinguishing between severe and non-severe COVID-19 cases [13]. Our findings reveal a lower Gal-3 threshold of 13.47 ng/ml, demonstrating greater sensitivity than the higher cutoff values reported in other studies [14]. This variation, reflecting differences in clinical practices, demographics, and patient health statuses, underscores the need for standardized research methodologies and larger, diverse patient cohorts to to utilize Gal-3 across various clinical settings effectively. As COVID-19 transitions from a global health emergency, the evolving epidemiological landscape offers a unique opportunity to refine our understanding and management strategies through innovative molecular research, positioning Gal-3 not only as a diagnostic tool but also as a potential target for therapeutic interventions.

Recent research highlights the effectiveness of galectin-3 inhibitors, like ProLectin-M and GB0139, in reducing COVID-19 severity by lowering viral load and improving symptoms [15], showcasing their potential in combating virus-induced inflammation and pneumonitis. Furthermore, treatments targeting galectin-3 [16] and the mineralocorticoid receptor [17] have been shown to mitigate endothelial inflammation caused by the virus, pointing towards innovative strategies for treating COVID-19-related complications 17. As the role of galectin-3 as a biomarker becomes clearer, its clinical implications extend beyond prognostication to the potential for guiding therapeutic interventions. Given its ability to help identify high-risk patients, galectin-3 could assist clinicians in prioritizing treatment options for those with elevated levels, particularly as further research validates these findings. The integration of galectin-3 into routine clinical practice will require standardized measurement protocols and the development of safe, effective Gal-3 inhibitors.

By expanding on the link between irisin levels and severe COVID-19 prognosis, particularly in patients with diabetes and obesity [18], our study supports emerging research indicating irisin plays a significant role in combating COVID-19. Research showing the modulation of genes linked to severe COVID-19 outcomes in adipocytes by irisin highlights its potential as a biomarker [19], especially given the worsened clinical progression observed in diabetic and obese populations compared to individuals with no such conditions. However, the findings of our study lacked statistical significance, possibly due to the small patient sample size. This limitation emphasizes the need for further large-scale investigations to establish irisin’s prognostic utility in severe COVID-19 cases. The anti-inflammatory effects of irisin, emphasized in multiple studies, highlight its therapeutic potential in reducing inflammation-driven complications in severe COVID-19 cases [19], presenting a promising avenue for future treatment strategies. This underscores the necessity for additional research to thoroughly determine the prognostic value of irisin in severe COVID-19 cases, particularly considering its potential to affect outcomes in patients with underlying metabolic disorders.

### Limitations

- Small sample size and single-center design, which may limit the generalizability of our findings and reduce the statistical power to detect significant differences.
- Potential discrepancies in galectin-3 cutoff values, requiring further research to establish more definitive thresholds for clinical application.
- Variations in patient demographics and clinical practices may not be fully captured, impacting the overall applicability of results.
- As the epidemiological situation changes and severe COVID-19 cases decline, future research on the prognostic value of galectin-3 may need to adjust.

Larger, multi-center cohort studies are essential for validation, although laboratory-based or experimental models simulating viral infection could provide additional insights.

## Conclusion

The significant role of galectin-3 in COVID-19 pathophysiology highlights its utility as a biomarker and therapeutic target. Addressing the variability in biomarker thresholds and expanding our understanding through larger, diverse studies will be essential for enhancing clinical outcomes in severe COVID-19 cases.

## Data Availability

No datasets were generated or analysed during the current study.
